# Retrospective Survey of Human Trichinellosis in a Romanian Infectious Diseases Hospital over a Thirty-Year Interval—The Never-Ending Story

**DOI:** 10.3390/pathogens12030369

**Published:** 2023-02-23

**Authors:** Mihaela Lupșe, Angela Monica Ionică, Mirela Flonta, Mihai Aronel Rus, Violeta Briciu

**Affiliations:** 1 Clinical Hospital of Infectious Diseases of Cluj-Napoca, 400348 Cluj-Napoca, Romania; 2 Department of Infectious Diseases, “Iuliu Hațieganu”, University of Medicine and Pharmacy, 400348 Cluj-Napoca, Romania

**Keywords:** trichinellosis, clinical manifestations, complications, treatment

## Abstract

Trichinellosis remains a food-safety risk in Romania due to cultural traditions and food behavior. The aim of the present study was to evaluate the epidemiological, clinical and therapeutical data of all human trichinellosis cases in patients admitted to an Infectious Diseases Hospital from northwestern Romania during a thirty-year interval. Between 1 January 1988 and 31 December 2018, a total of 558 patients were hospitalized with the diagnosis of trichinellosis. The number of cases/year varied between 1 and 86. The source of infection was known for 524 patients, represented by domestic pig meat (n = 484; 92.37%) and wild boar (n = 40; 7.63%). Most patients (410; 73.48%) presented were part of family or group outbreaks. Demographical and clinical data of patients will be presented. Antiparasitic therapy was prescribed in 99.46% of cases, and corticosteroids were prescribed in 77.06% of patients. In total, 48 patients (8.6%) presented complications of trichinellosis: 44 for a single complication (neurological, cardiovascular or respiratory); the others multiple complications. Pregnancy was documented in five patients. No fatalities occurred during the study period. Although the number of hospitalized patients has decreased in the last years, trichinellosis still remains an important public health problem in northwestern Romania.

## 1. Introduction

Nematodes of the genus *Trichinella* are amongst the most widespread foodborne zoonotic pathogens, with infections having been diagnosed in numerous species of domestic and wild animals on all continents except for Antarctica [1]. According to the United Nations Food and Agriculture Organization (FAO) and World Health Organization (WHO), infection by *Trichinella spiralis* was classified as the seventh most important food-borne parasite of global concern [2]. Based on genetic studies, eight species and four additional genotypes have been recognized [3]. 

Contamination of the vertebrate host occurs by ingestion of meat infected with first-stage larvae (L1), which are released during gastric digestion and invade the small bowel mucosa [4]. As early as day 5 postinfection, the females begin releasing larvae, which migrate throughout the host’s blood and lymphatic vessels, taking up residence in the striated muscle cells [4,5]. The larvae cause major perturbations of the host cells, where they encapsulate [6]. The survival of the larvae is variable, but it was noted that it can last for up to 40 years in humans [7].

Although *Trichinella* spp. are able to infect a large variety of vertebrate species, it is believed that only humans become clinically affected. The severity of the disease, however, is largely dependent on the number of ingested larvae and can range from asymptomatic to fatality [8]. Trichinellosis in humans is strictly related to cultural food practices, including the consumption of raw or undercooked meat of different animal origin [9]. The most important source of human infection is represented by the domestic pig, particularly in areas where the pigs are raised in free-range or backyard conditions. Other significant species during outbreaks in Europe are represented by horses and wild boars [8].

Recent data indicate that during 2013–2020, a total number of 1242 cases of human trichinellosis were reported in the European Union, with 497 cases officially reported in Romania, meaning a local incidence ranging between 0.02 and 1.11 cases per 10^6^ inhabitants per year [10,11]. In the latest report of the National Institute of Public Health on transmissible diseases in surveillance, 58 cases of trichinellosis were reported in 2020 [12]. However, the actual frequency of the disease may be underestimated, due to misdiagnosis, as clinical manifestations may overlap those of other diseases, such as respiratory disease, gastroenteritis or skin allergies [13,14].

In Romania, thus far, both *T. spiralis* and *T. britovi* have been identified in domestic pigs and a variety of wildlife hosts, as reviewed by Gherman et al. [15]. It seems that infections in humans caused by *T. spiralis* are generally more severe as compared to those caused by *T. britovi*, possibly because females of *T. britovi* are less prolific [16].

The clinical course of the acute infection is characterized by diarrhea, anorexia, vomiting, abdominal pain during the enteral phase, and facial oedema, muscle pain and swelling, weakness, fever, anorexia, headache, conjunctivitis, and urticaria in the systemic phase [17]. Dyspnoea is caused primarily by parasite invasion and inflammation of respiratory muscles, such as the diaphragm, but bronchopneumonia and infarction may also occur [18]. Myocarditis is the most frequent cardiovascular complication, leading sometimes to heart failure and arrhythmias [19]. Neurological manifestations, more common in severe infections, occur in 0.2% to 52% of cases [20].

Current surveillance of trichinellosis in Romania is a passive surveillance, with mandatory declaration, according to national legislation, of each suspected or confirmed case, by any health care provider. The local public health department performs an epidemiological investigation for any case reported [21].

The aim of the study was to evaluate the epidemiological and clinical aspects and to assess therapeutic approaches of all human trichinellosis cases in patients admitted to an Infectious Diseases Hospital situated in the Transylvania region, northwestern Romania, during a 30-year interval, addressing the question of whether this zoonosis is still an important public health problem.

## 2. Materials and Methods

### 2.1. Study Design and Setting

A retrospective study on consecutive hospitalized patients was performed in The Clinical Hospital of Infectious Diseases Cluj-Napoca. The Clinical Hospital of Infectious Diseases Cluj-Napoca is an academic monospecialty hospital that provides medical services for patients with infectious pathology from Cluj County and neighbouring counties.

### 2.2. Participants

All patients discharged with the diagnosis of trichinellosis between 1 January 1988 and 31 December 2018 were included. The diagnosis of trichinellosis was established by patients’ current physicians (an infectious diseases specialist) based on epidemiological (exposure to contaminated food (meat), exposure to a common source), clinical and paraclinical data. Although not available in the hospital’s laboratory, serological tests (ELISA detection of IgM or IgG specific antibodies) were performed in some cases in external laboratories, prior to admission, and results were available in patients’ files.

### 2.3. Variables

The analysed data included demographic data (age, sex, urban/rural residency, county), epidemiological data (food source, date of consumption of *Trichinella* suspected or confirmed infected food, any other family member with suspected or confirmed trichinellosis), and clinical data: date of onset of clinical symptomatology, date of hospitalization and discharge, signs and symptoms at hospital admission (i.e., fever, fatigue, myalgia, muscles affected by pain, nausea, vomiting, diarrhea, abdominal pain, headache, conjunctival haemorrhage, peripheral oedema, periorbital oedema, rash, productive cough, dyspnoea, retrosternal pain) were recorded. Laboratory data collected were: complete blood count (CBC) and the presence of IgM or IgG antibodies against *Trichinella spiralis* whenever available. Based on the CBC, eosinophilia was classified as: mild (500 to 1500/μL), moderate (1500 to 5000/μL), and severe (>5000/μL) [22].

Data on antiparasitic drugs used for therapy, corticotherapy, and the duration of treatment were recorded. 

Complications of trichinellosis were classified as: myocarditis, respiratory failure, and CNS manifestations. The diagnosis of myocarditis was confirmed by the cardiologist, as general practice is used to screen systematically (even when specific symptoms are missing) and diagnose cardiac involvement based on clinical symptomatology, ECG abnormalities, modification of cardiac markers (increased troponin level) and consultation with a specialist in cardiology (as well as echocardiogram in selected cases)**.** Respiratory failure was defined clinically by the presence of dyspnoea and decreased peripheral oxygen saturation (<93% in room air). The presence of CNS manifestations was recorded based on the diagnosis at discharge, by the attending physician, the infectious diseases specialists, and the diagnosis found in the medical file of the patients.

### 2.4. Statistical Analysis

Categorical data were presented as counts and percentages. Comparisons between two independent groups concerning categorical data were performed with chi-squared test or Fisher exact tests (in case of low expected frequencies). Correlations were assessed using Pearson’s test. Means were compared by means of ANOVA.

## 3. Results

**Figure 1:**
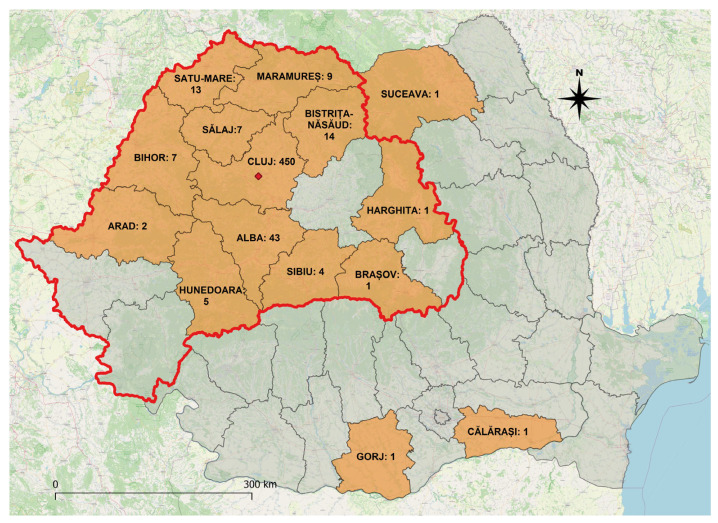
Map of Romania representing patient residence. The location of the hospital is marked with a red dot. Number of hospitalized patients from each county is included on the map. Transylvania region is outlined in red.

During the study interval, a total of 558 patients were discharged having the diagnosis of trichinellosis. Most of the patients (80.6%) originated from Cluj County, where the hospital is located, the others having residence in 14 counties, mainly the Transylvania region (Figure 1).

**Figure 2:**
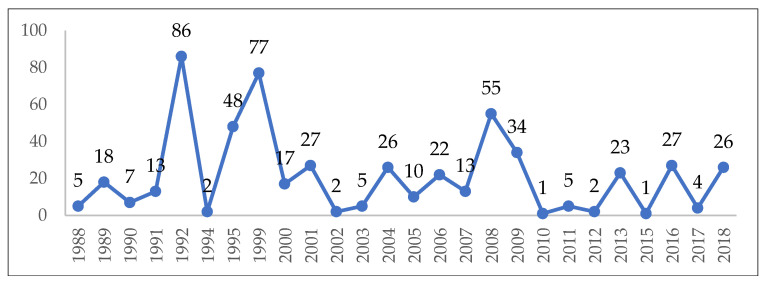
The annual distribution of admitted trichinellosis cases.

The number of cases hospitalized per year varied between 1 and 86, with annual fluctuations (Figure 2). During the study interval, the highest number of cases were diagnosed in the month of January (n = 271; 48.57%), followed by February (n = 95; 17.03%), while 63 cases were recorded during summer/autumn months (June–November).

The distribution of study group according to gender and residency is presented in Table 1.

The age of the hospitalized patients ranged between 11 months and 87 years, with a medium value of 34.01 ± 17.49 and a median of 34 years. The distribution of patients according to ECDC age group categories is presented in Table 2.

The source of infection was known for 524 patients and was represented mostly by domestic pig meat and products (n = 484; 92.37%), while the rest (n = 40; 7.63%) were due to consumption of wild boar. The origin of the domestic pig was further detailed by 171 patients, of which 37 (21.64%) had bought the meat from unauthorized sources, while the rest (n = 134; 78.36%) reared the pigs themselves in backyard systems. The epidemiological investigation identified multiple consumers of the same source of infection, varying from 2 to 50 people. Over the study period, a total of 10 major clusters (including 10 or more consumers of the same source) were identified (Table 3). Among these, asymptomatic patients also presented for evaluation and treatment, due to the epidemiological link.

Data regarding the incubation period were recorded in the medical files of 219 patients, for whom the onset of clinical manifestations ranged between less than 1 day and 64 days, with an average of 16.43 ± 11.5 days and a median of 14 days, respectively. However, 58 patients (26.48% of the 219 subgroup) had a short incubation interval, presenting first symptoms in the first week following consumption of infected meat.

**Figure 3:**
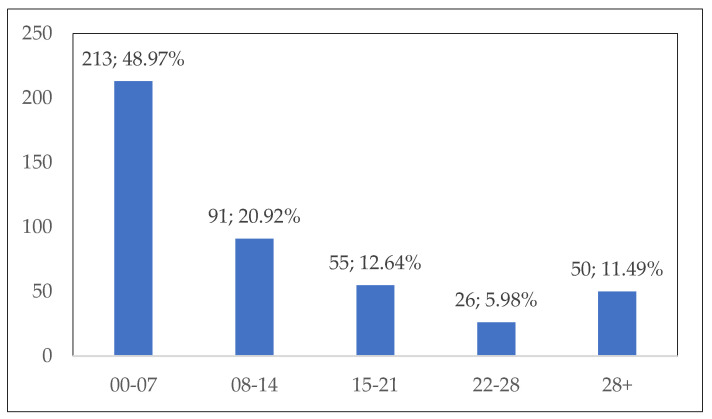
Interval (days) between the onset of clinical manifestations and hospital admission.

The interval between the onset of clinical symptoms and hospital admission was recorded for 435 patients, as illustrated in Figure 3. 

Forty-five patients (8.06%) were asymptomatic, while the rest exhibited between 1 and 11 symptoms at hospital admission, as detailed in Table 4. The number of symptoms was in a significant slight negative correlation (r = −0.103; *p* = 0.031) with the number of days spent between the onset of clinical symptoms and hospital admission. There was no significant correlation between the incubation period and the number of displayed symptoms (r = 0.037; *p* = 0.582). 

The most frequent symptom (n = 424; 75.99%) was myalgia, affecting one or several muscle groups. Overall, the most frequently affected regions were the lower limbs (n = 261; 61.56%), followed by the upper limbs (n = 198; 46.69%), the thorax (n = 158; 37.26%), and the lumbar region (n = 147; 34.67%). Myalgia of the neck muscles and masseters was reported by 18 (4.24%) and 11 (2.59%) patients, respectively. The frequency of myalgia was significantly lower (*p* < 0.001) in children (46.94%) than in adults (82.17%). Similarly, a significantly lower proportion of children (*p* < 0.001) presented periorbital oedema (26.53%), fever (28.57%), or fatigue (25.51%), as compared to adults (59.13%, 51.30%, and 46.96%, respectively).

**Figure 4:**
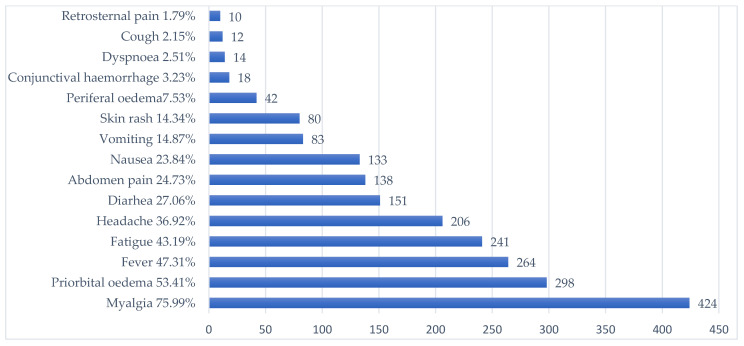
The frequency of signs and symptoms in trichinellosis patients.

The presence of other symptoms and signs, according to frequency, is represented in Figure 4.

The diagnosis was confirmed by external laboratories, prior to admission, by detection of IgM and/or IgG antibodies in 36 (6.45%) of the patients, with IgM positive in 3 patients and IgG positive in 33 patients. The CBC performed at admission indicated hypereosinophilia of various degrees in 419 patients (75.09%), as follows: 203 mild, 183 moderate, and 33 severe. The eosinophil counts varied between 10 and 19,700/μL, with an average of 1789, and a median of 1140. The average eosinophil counts were significantly (*p* < 0.001) lower in children (1072/μL) as compared to adults (1942/μL). The CBC indicated an elevated number of leucocytes (>10,000/μL) for 161 (27.38%) of the admitted patients. The values varied between 3100 and 41,200/μL, with an average of 9395/μL, and a median of 8200/μL, and there were no statistically significant differences (*p* = 0.569) between children (average 8667/μL) and adults (average 9552/μL). 

The duration of hospitalization varied between 1 and 41 days, with an average of 8.46 ± 4.65 days and a median of 8 days.

The antiparasitic treatment included mainly benzimidazoles (albendazole, mebendazole, thiabendazole) (97.85%), piperazine, and diethylcarbamazine. In some patients, treatment was initiated with one regimen and continued with another one as presented in Table 5. The duration of antiparasitic treatment ranged between 1 and 43 days, with an average of 10.4 days and a median of 11 days. 

Corticosteroids were administered to 430 patients (77.06%). In some patients, a corticosteroid product was changed to another during the hospitalization (Table 6). The mean eosinophil counts were significantly higher (*p* < 0.01) in patients who received corticosteroids (2157.6/μL), as compared to those who did not (1168.7/μL). For patients who received corticosteroids, the duration of hospitalization ranged between 1 and 41 days, with an average of 9.1 days and a median of 8 days, while for patients who did not receive corticotherapy, the duration ranged between one and 14 days, with an average of 6.3 days and a median of 6 days. The number of hospitalization days in patients treated with different corticosteroid products is detailed in Table 6.

A total of 48 patients (8.6%) presented complications of trichinellosis. Among them, 44 presented a single complication: 17 CNS manifestations (3%), 15 myocarditis (2.6%), and 12 respiratory failure (2.1%). The others presented more than one complication as follows: two patients presented myocarditis and CNS manifestations, one had respiratory failure and myocarditis, and one presented all of the three complications. 

During the study period, five cases of pregnant women were recorded: two with residency in rural areas and three in urban areas. The patients’ ages varied between 19 and 37 years old. The number of recorded symptoms ranged between four and nine. Myalgia of the lower limbs and upper limbs and periorbital oedema were present in all cases. Four of the patients presented headaches, and two presented fever. The CBC revealed mild and moderate eosinophilia for two patients. Two patients did not receive antiparasitic medication, while three were treated as follows: mebendazole followed by diethylcarbamazine for 14 days; mebendazole for 12 days; thiabendazole followed by diethylcarbamazine for 9 days. Two patients received prednisone, two received hydrocortisone hemisuccinate, and one received hydrocortisone hemisuccinate changed to dexamathazone. The duration of hospitalization varied between 4 and 14 days. One of the pregnant patients, a 37-year-old woman, hospitalized in 1990, presented all three complications: CNS manifestations, myocarditis, and respiratory failure. She was treated with mebendazole and prednisone and discharged in 12 days.

No fatalities occurred during the study period.

## 4. Discussion

Globally, between 1986 and 2007, a total of 65,818 cases and 42 deaths were reported from 41 countries, most of which (56,912) occurred in Europe, particularly in Romania (28,564), mainly during 1990–1999 [23]. The European Union (EU) reported 5518 trichinellosis cases during 2002–2017, indicating that the prevalence of the disease is decreasing. Furthermore, almost half of the EU countries did not have any documented trichinellosis infections during this time span [23]. In Romania, between 1979 and 2007, the overall incidence of human trichinellosis was 4.5 cases per 10^6^ inhabitants per year, with significant local and annual variations, as reviewed by Neghina et al. [24]. Between 1980 and 2004, the incidence rate obtained for the Transylvania region was higher (82.2 cases per 10^6^ persons per year) than the incidence rate obtained for the other counties (35.7 cases per 10^6^ persons per year), in relation to the tradition of consuming products obtained from raw pork meat [25], despite the specific national and European legislation regarding meat inspection. This is mainly a consequence of the lack of public awareness and poor sanitary education—the sanitary measures being often regarded as abusive and unnecessary. A global decrease in incidence has been noted, but the trend seems to be shifting frequently. In our study group, the highest number of hospitalized patients was in 1992, followed by the year 1999. The first peak in hospitalization corresponds to a nation-wide increase in cases, as a result of political changes, associated with a decrease in the efficacy of food safety control [25]. The phenomenon of “urbanization” of trichinellosis was also observed during this period, with a high proportion of cases being diagnosed in urban areas [24], as was also noted during the present study. While most residents of the rural areas raise their own pigs in backyard systems (with varying degrees of compliance to the applicable hygienic rules), the urban population is used to buying pigs from animal markets, immediately transporting them for household slaughtering in the countryside (at friends or relatives). That may explain the higher percentage of females from urban areas in our cohort of patients, especially as women are mostly involved in preparing meat products and often taste the raw meat during the process. 

Most of the cases were diagnosed in the month of January, followed by February. This is mainly explained by one of the most important winter traditions in Romania, the tradition of pig slaughtering and consuming pork meat and derived products (such as smoked raw sausages) during winter months (especially around Christmas). More than half of the patients (410; 73.48%) presented as part of family or group outbreaks. This may be due to the tradition known as “pig’s alms”, representing the thanksgiving meal offered to relatives, friends, or neighbours who participated in the pig slaughtering process [26]. In a European Union report on trends and sources of zoonoses, zoonotic agents and food-borne outbreaks in 2017, Romania accounted for most positive fattening pigs that were not raised under controlled housing conditions [27]. The seasonality of trichinellosis was also partly in relation with the wild boar hunting season, which in Romania starts on the 1st of June and lasts until the 31st of January, as 40 patients (7.17%) of our study group were infected due to consumption of wild boar.

The disease incubation period ranges from 7 to 30 days, related to the number of larvae ingested, which, in turn, usually determines the severity of disease [20]. The average incubation period during the present study, 16.43 days, was longer than reported in a case series from Western Romania, which was of 7.6 days, with 81.9% of patients presenting clinical symptoms within the first 10 days postinfection [28]. 

The interval between the onset of clinical symptoms and hospital admission was longer than seven days in more than half of the patients. These data suggest a delay in diagnosis, possibly due to misdiagnosis, as suggested by Nemet et al. [14], or due to moderate symptomatology, ignored or self-medicated by the patient. Delayed diagnosis and prescription of an inappropriate treatment may result in severe disease course and complications. Although no pathognomonic signs and symptoms exist for trichinellosis, in our study group, more symptoms were associated with a shorter interval between clinical onset and hospitalization. An important percentage of patients (11.49%) were hospitalized more than 28 days after the onset of symptomatology. The recorded clinical manifestations at admission suggest that most patients presented to the hospital during the systemic phase of the disease. Localized or general myalgia was the most common manifestation of all symptomatic patients, and muscles of the lower limbs were the most frequently affected, most likely due to the high level of activity and oxygenation [4]. Other frequently encountered symptoms consisted in periorbital oedema, fever, fatigue and headache. Apart from periorbital oedema, these symptoms are nonspecific and are associated in the clinical picture of a variety of infectious diseases, thus complicating the differential diagnosis of trichinellosis [14]. Especially during the cold season, trichinellosis is associated with a high incidence of viral infections. Periorbital oedema, although an alarm sign for trained physicians, might be overlooked by doctors that have never seen a trichinellosis patient. Increasing awareness in medical communities in both endemic and non-endemic regions might improve the chance of rapid suspicion and diagnosis of trichinellosis. Within the same cluster of infection, a variation in the clinical presentation of patients was noted, possibly as a consequence of the amount of larvae ingested.

Children are also affected by trichinellosis, but myalgia, facial oedema and eosinophilia were less common than in adults—findings confirmed also by other studies [29]. A smaller amount of meat consumed, with lower number of ingested larvae, or well-done meat served to children might be the explanation. In our study group, trichinellosis was diagnosed even in one infant, underlining parents’ lack of education regarding food-borne infections.

In clinically manifest trichinellosis, leucocytosis accompanied by eosinophilia will usually occur as a nonspecific laboratory marker [8]. It has been suggested that muscular damage may be mediated indirectly by activated eosinophils, sustained by numerous observations that eosinophilia is correlated with the degree of myalgia [13]. In the present study, the intensity of myalgia was not scored. Hypereosinophilia of up to 19,000/μL is reported by Bruschi et al. as a laboratory finding in human trichinellosis [21]. Most patients presented hypereosinophilia, including one patient with a value of 19,700/μL, who presented a mild form of the disease, with no complications. A sudden reduction in the level of circulating eosinophils to 1% or none is an indication of severe infection and may even signal the onset of death of the patient [17]. Fourteen of the patients (2.51%) in our study presented eosinophil level less than 100/μL, with the minimum value of 10/μL, and complications were described in two of them. In one patient, with an eosinophil level of 84/μL, CNS manifestations were described, while in the pregnant woman having 100 eosinophils/μL, all three considered complications were present. Hyperleucocytosis was present in 27.38% of the admitted patients, while in the study on 83 patients from western Romania, 51.8% had high levels of white blood cells (more than 10,000/mm^3^) [28]. Hyperleucocytosis with values up to 41,200/μL, as seen in our patients, may alert the physician for differential diagnosis with leukemoid reactions [29]. 

The length of hospitalization might be used as an indirect measure of disease severity. In the present study, the mean duration of hospitalization was 8.46 days, close to that reported by Lupu et al. [28], at 7.5 days.

The detection of specific anti-*Trichinella* antibodies in blood serum is of great diagnostic value, included in the laboratory criteria of confirmed trichinellosis used in the EU, according to the commission implementing decision (EU) 2018/945 of 22 June 2018 [30]. Access to immunological assays was not easily available during the 30-year-study interval. IgM and/or IgG anti-*Trichinella* antibodies were recorded for 36 (6.45%) of the patients. The rest of the patients from our study group could be classified as probable trichinellosis based on the 2018 case definition (clinical criteria and epidemiological link) [31]. The presence of IgG specific antibodies could be explained by the late presentation. The time of seroconversion after a primary *Trichinella* infection is dependent upon the infection dose and the larval burden in the muscle. Following the ingestion of high numbers of *T. spiralis* larvae, anti-*Trichinella* immunoglobulin G (IgG) can be detected in animals about 2 to 3 weeks postinfection [8]. Longitudinal studies revealed that anti-*Trichinella* antibodies may persist in pigs for a long and presumably indefinite time [32]. Not enough information exists on the protective immunity of these IgG antibodies, and questions exist on the possible risk of reinfection in persons that continue to have exposure by culinary habits. Although reinfection with *T. spiralis* occurs frequently in domestic and wild animals, the host responses to reinfection with *T. spiralis* remain largely unknown. A study on kinetics of immunological response and resistance to reinfection on mice showed that resistance was present four weeks after the primary infection and was maintained for the duration of the study [33]. Another study on mice investigated infective dose on primary infection that induces significant resistance against reinfection, closely correlated with *T. spiralis*-specific IgG [34]. We cannot exclude the possibility of persistent IgG antibodies in persons with previous exposure, undiagnosed trichinellosis and reinfection with a milder disease. Further studies should address this question of reinfection in humans, especially in highly endemic areas and populations at risk.

The treatment goal for the very early infection phase is to limit muscle invasion by larvae; when this has already occurred, the goal is to reduce muscle damage, which is responsible for the major clinical manifestations. The administration of efficacious anthelmintic drugs at the stage of intestinal invasion or in the acute phase is crucial for effective therapy. The later a treatment is prescribed, the greater the probability that the infected person will already harbour viable larvae in their muscles, which can then survive for years despite treatment, with possible persistent myalgia [35]. Anthelmintics, primarily albendazole and mebendazole, are the principal drugs for the treatment of trichinellosis. Thiabendazole is less frequently used because of its side effects [36]. Diethylcarbamazine is an anthelmintic used to treat filarial infections [37]; it was used as single therapy in nine patients, and for continuation of benzimidazole treatment in 56 patients (Table 4). That treatment was used mainly in the first 11 years of our study, between 1988 and 1999, due to a lack of treatment consensus in trichinellosis. Interests in treatment with diethylcarbamazine of other nematode infections except for filarial diseases previously existed [38], and that might explain its clinical use in trichinellosis patients from our cohort. Piperazine is used as an alternative treatment for ascariasis and enterobiasis, and its indication for trichinellosis has never been established [39]. We found its use in seven of our study group patients, all treated between 1992 and 1999. Monotherapy with benzimidazole was prescribed in 78.26% of our cohort. We did not find information on the medical decision for change in antiparasitic therapy from one drug to another, but the administration of different antiparasitics in 109 (19.64%) of our treated patients supports the lack of consensus due to limited information and possible lack of supply of different products. Lack of consensus on product was accompanied by a lack a consensus on the duration of treatment. The recommended duration for albendazole is 8–14 days, and for mebendazole is 13 days [40], but in our study group, 29 (5.2%) patients received antiparasitic therapy for more than 14 days, with an extreme of 43 days. The Center for Disease Control, United States of America, recommends that if case treatment is not initiated within the first several days of infection, more prolonged or repeated courses of treatment may be necessary, without a specific indication [41]. In conclusion, uncertainty on the ideal antiparasitic and duration of treatment for trichinellosis exists. Even at the present time, albendazole and mebendazole are not FDA-approved for trichinellosis treatment [42]. According to WHO guidelines for mass prevention campaigns, albendazole can be used in children as young as one year old [43]. In our study group, all children treated with albendazole were older than one year. 

Anthelminthics must always be given before corticosteroids to prevent the effect of delayed expulsion of adult worms from the intestines [44]. Prednisone is given with anthelminthic drugs to prevent worsening symptoms and to shorten the symptomatic period [45]. Corticosteroids are recommended to suppress the vascular and muscular damage induced by eosinophils [46]. Their administration in patients with severe trichinellosis shortens the course of the illness [44]. Following the implementation of corticotherapy, the rate of fatality in neurotrichinellosis significantly decreased from 46% to 17% [20]. In our study group, corticosteroids were prescribed in 77.06% of patients. In a review on trichinellosis [8], corticosteroids are recommended in severe and moderately severe disease, while nonsteroidal anti-inflammatory drugs, if necessary, in benign, abortive, and asymptomatic disease. We did not perform in clinical practice a classification on dependence of the severity of signs and larval density, as proposed by Gottstein et al. [8], but the decision of glucocorticoid therapy and duration of treatment was made by the infectious disease specialist in each patient based on clinical severity judgement. The decision was also supported by the severity of eosinophilia. In some patients, a corticosteroid product was replaced by another product during hospitalization, with the aim of changing intravenous administration to oral administration or to decrease the number of doses/day. 

In our study group, we have further investigated the evolution of complications in trichinellosis. As the presence of central nervous system (CNS) manifestations was recorded in the study database from patient files, as established by the current physician, we could not evaluate the diversity and severity of these manifestations. Neurological involvement of trichinellosis generally occurs in the most severely affected patients. *Trichinella* larvae can migrate in the CNS and cause diffuse lesions, obstruction of the blood vessels, and inflammatory infiltrate [47]. A recent review on 162 neurotrichinellosis cases identified headache in 24.69% of patients, confusion in 14.2%, disorientation in 11.73%, delirium in 9.87% and meningeal signs in 14.8% [48]. Central motor deficits presented as hemiparesis (19.14%) or tetraparesis (10.49%), while cognitive problems included memory impairments, mainly affecting recent memory, aphasia, acalculia, apraxia and anosognosia, which were described in less than 10% of the patients [48]. 

In total, 19 (3.4%) of our cohort presented cardiac complications. Myocarditis is the most common cardiac complication of trichinellosis, but pericarditis, endocarditis, vascular thrombosis, or embolism may also be diagnosed. It has been noted that ECG anomalies represent the most common signs of cardiac involvement in nonfatal cases and generally occur during the second week of illness [19]. We have not recorded the type of cardiac involvement separately.

Fourteen (2.5%) of our cohort of patients presented respiratory failure as a complication of trichinellosis. Respiratory symptomatology during cold season, such as productive or dry cough, dyspnoea associated with fever, myalgia, headache and fatigue, is a frequent cause of misdiagnosis in trichinellosis. A study from Central Romania evaluating 699 cases of trichinellosis identified misdiagnosis in almost half of the cases, with a third of misdiagnoses being attributed as respiratory pathology [14]. Respiratory complications were described in 1% of the same study group population [49]. Acute respiratory distress syndrome, with pulmonary oedema and need for mechanical ventilation, was previously reported by the WHO in severe trichinellosis [50].

All three severe trichinellosis complications were associated with one pregnant woman, out of the five included in our study. Worldwide, an important number of trichinellosis outbreaks involving pregnant women have occurred over time. Little is known about the consequences of this infection in the course of pregnancy, as well as its impact on the embryo/foetus development. Although theoretically possible, vertical transmission of *Trichinella* spp. has rarely been investigated. A study on six pregnant women suggests that transplacental passage of new-born larvae is possible [51]. A case report from The Slovak Republic confirmed *Trichinella* larvae that infected the foetus in the early stage of development [52]. Both mebendazole and albendazole are relatively contraindicated in pregnancy; however, three out of five pregnant patients consented to treatment with mebendazole, thiabendazole or diethylcarbamazine, because of severe symptomatology and concern that foetal infection might lead to severe complications. No information on pregnancy and foetal outcome is available. 

No fatalities occurred during the study period, which represents a major achievement on the management of the 558 hospitalized patients over a period of thirty years. Worldwide, from 1986 to 2009, from the 65 818 trichinellosis cases reported, 42 deaths were recorded [53], indicating a relative low morality rate.

### Limitations and Strengths

This study presents data on human trichinellosis from a large cohort of 558 patients hospitalized in an infectious diseases hospital over a period of thirty years. Although previous data have been published on hospitalized patients from western [30] and central Romania [14,49], until now, no data were available regarding epidemiological and clinical features, as well as therapeutical management of patients from northwestern Romania. A limitation of the study is represented by the absence of data intended for collection in some medical files, explained by the retrospective nature of the study over a long interval. 

Although our thirty-year study does not bring any new clinical data on human trichinellosis or particularities in investigated patients, nor case management discordances with current recommendations, it highlights the continued risk represented by this neglected food-borne infection with the aim of increasing awareness in the medical and scientific communities and pointing out that trichinellosis is not a forgotten disease. 

## 5. Conclusions

Public health awareness, continuous monitoring of meat sources and improved access to diagnosis still represent the goals to reducing the risk of human trichinellosis worldwide. As with other foodborne zoonoses, cultural traditions in food behaviour and practices in the use of domestic and wild animals are not easily altered, and trichinellosis can be expected to remain a food-safety risk and an important health problem in Romania in the future.

## Figures and Tables

**Table 1:** The distribution of patients according to sex and residency.

	Urban		Rural		Total	
Male	138	41.32%	106	47.32%	244	43.73%
Female	196	58.68%	118	52.68%	314	56.27%
Total	334	59.86%	224	40.14%	558	100%

**Table 2:** The distribution of patients according to age groups.

Group (Years)	n	%
00.0–04.9	27	4.84
05.0–14.9	46	8.24
15.0–24.9	111	19.89
25.0–44.9	215	38.53
45.0–64.9	135	24.19
65.0 +	24	4.3

**Table 3:** Major trichinellosis clusters identified during the study period.

Year	Month	Source	Consumers *	Admitted	Symptoms									
					0	1	2	3	4	5	6	7	8	9
1995	09	boar	50	12	10	2	-	-	-	-	-	-	-	-
1992	01-02	pig	38	24	3	-	2	6	7	3	2	-	1	-
1995	01-02	pig	unspecified	19	-	1	3	4	4	5	1	1	-	-
2006	01	pig	20	19	2	3	7	2	3	-	1	1	-	-
2000	12	pig	20	15	2	3	2	5	2	1	-	-	-	-
1992	01-02	pig	18	17	-	-	1	2	4	5	1	1	3	-
2018	01	pig	16	8	-	1	3	2	1	1	-	-	-	-
1999	01	pig	15	13	-	-	2	1	-	3	2	3	1	1
2004	01-02	boar	13	8	-	-	-	2	3	2	1	-	-	-
2009	01-02	pig	unspecified	10	-	1	1	4	3	1	-	-	-	-

* as resulted from the epidemiological enquiry.

**Table 4:** Number of symptoms at hospital admission.

Symptoms	Children		Adults		Total	
	n	%	n	%	n	%
0	6	6.12	39	8.48	45	8.06
1	2	2.04	32	6.96	34	6.09
2	16	16.33	52	11.30	68	12.19
3	18	18.37	85	18.48	103	18.46
4	17	17.35	86	18.70	103	18.46
5	15	15.31	74	16.09	89	15.95
6	8	8.16	49	10.65	57	10.22
7	8	8.16	24	5.22	32	5.73
8	4	4.08	11	2.39	15	2.69
9	3	3.06	7	1.52	10	1.79
10	0	0	1	0.22	1	0.18
11	1	1.02	0	0	1	0.18

**Table 5:** Administered antiparasitics and the duration of treatment.

Antiparasitic	Duration of Treatment (Days)				Total	
	0–9	10–14	>14	No Data	n	%
albendazole	42	195	18	10	265	47.49
mebendazole	106	19	0	43	168	30.11
mebendazole/diethylcarbamazine	2	30	2	6	40	7.17
albendazole/mebendazole	14	14	2	5	35	6.27
thiabendazole/diethylcarbamazine	6	4	3	1	14	2.51
thiabendazole/mebendazole	7	0	2	2	11	1.97
diethylcarbamazine	3	6	0	0	9	1.61
piperazine/mebendazole	5	2	0	0	7	1.25
thiabendazole	3	1	0	0	4	0.72
mebendazole, thiabendazole, diethylcarbamazine	0	0	2	0	2	0.36
no treatment	0	0	0	0	3	0.54
Total	188	271	29	70	558	100

**Table 6:** Administered corticosteroids and hospitalization days in relation to used corticosteroid therapy.

Corticosteroid	n	%	Hospitalization Days			
			Minimum	Maximum	Average	Median
hydrocortisone hemisuccinate	162	29.03	1	17	7.1	6
prednisone	153	27.42	2	36	10	9
dexamethasone	71	12.72	2	20	10.4	10
hydrocortisone hemisuccinate/prednisone	24	4.30	3	34	10.6	9
hydrocortisone hemisuccinate/dexamethasone	8	1.43	4	41	10.4	5
dexamethasone/prednisone	7	1.25	4	16	12.7	14
treated, no data on product	5	0.90	8	13	10.8	11
no corticotherapy	128	22.94	1	14	6.3	6

## Data Availability

The complete dataset used and analysed during the current study areavailable from the corresponding author on reasonable request.
